# Immune Response and Lipid Metabolism Genes Associated with Acute Cerebral Circulatory Failure in Pre-Eclampsia in a Central Asian Population

**DOI:** 10.34763/jmotherandchild.20252901.d-25-00019

**Published:** 2025-11-14

**Authors:** Dinara Mirzakhmetova, Gulnara Svyatova, Galina Berezina, Alexandra Murtazaliyeva

**Affiliations:** Scientific Center of Obstetrics, Gynecology and Perinatology, 050020, 125 Dostyk Ave., Almaty, Republic of Kazakhstan; Research and Education Center, Center for Molecular Medicine, 050026, 130 Aitiev Str., Almaty, Republic of Kazakhstan; Department of Continuing Education, Scientific Center of Obstetrics, Gynecology and Perinatology, 050020, 125 Dostyk Ave., Almaty, Republic of Kazakhstan; Republican Medical Genetic Consultation, Scientific Center of Obstetrics, Gynecology and Perinatology, 050020, 125 Dostyk Ave., Almaty, Republic of Kazakhstan

**Keywords:** Single nucleotide polymorphisms, vascular complications, lipid metabolism, immune markers, genetic risk factors, endothelial dysfunction

## Abstract

**Background:**

The aim of this study was to determine the association of immune response and lipid metabolism genes with the development of pre-eclampsia and related acute cerebral circulatory disorders in Kazakh women.

**Materials and methods:**

Minor allele frequencies of immune response and lipid metabolism genes were determined in 1,800 healthy participants stored in the Miras Biobank of the Scientific Centre of Obstetrics, Gynaecology, and Perinatology Joint Stock Company.

**Results:**

The main results of the study showed that TLR4 (rs4986790), PLEKHA1 (rs2281673), PLEKHG1 (rs9478812), APOE (rs7412), FTO (rs1421085) and LPL (rs285) genes were in genetic equilibrium (p > 0.05), indicating that the sample was representative and there were no significant evolutionary pressures on the studied genes. The frequencies of minor alleles in the studied sample of Kazakhs were: TLR4 – 3.3%, PLEKHA1 – 5.9%, PLEKHG1 – 27.0%, APOE – 7.8%, FTO – 28.3% and LPL – 36.0%.

**Conclusion:**

The obtained data can be used for further genetic studies, as well as for the development of individualised strategies for the prevention and treatment of pre-eclampsia and related complications. The identified genetic markers may help in early detection of women at increased risk of developing these conditions, which will contribute to improving clinical outcomes and reducing maternal and perinatal mortality.

## Introduction

Acute cerebrovascular disorders (ACVD) are a major cause of global mortality and disability, with a significant impact on public health and the economy. The significance of this study is dictated by the fact that pre-eclampsia, a serious complication of pregnancy, significantly increases the risk of vascular disease, including stroke, in women of reproductive age. Pre-eclampsia significantly increases the risk of vascular complications, such as strokes, especially in women with the unique genetic background of the Kazakh population. The problem is the lack of understanding of genetic factors related to immune response and lipid metabolism, which hinders the development of personalised strategies for prevention and treatment of these conditions. In 2019, pre-eclampsia occupied 12.9% of all critical obstetric conditions in the city of Almaty (Kazakhstan) [1]. In conformity with the opinion of D. Zheng et al. [2], pre-eclampsia significantly increases the risk of ACVD both during pregnancy and after delivery. In their study, D. A. Mitrokhin et al. [3] state that eclampsia, characterised by high blood pressure and convulsive seizures, significantly increases the risk of ACVD. Women with eclampsia are at higher risk of developing both ischaemic and haemorrhagic stroke in the postpartum period. In their study, a prevalence of ischaemic stroke (64.7%) over haemorrhagic stroke (35.3%) was found when looking at the outcomes of 17 women aged 19 to 39 years who had a postpartum ACVD between 2017 and 2021.

Genetic studies have revealed that genes responsible for immune response and lipid metabolism may be crucial in the development of ACVD. These genes influence inflammatory processes and lipid metabolism, which in turn may contribute to vascular complications, including ACVD. In particular, abnormalities in immune response genes can lead to an increased inflammatory response, which enhances vascular damage and increases the risk of thrombosis. Also, alterations in genes regulating lipid metabolism can lead to dyslipidaemia, which is a significant risk factor for atherosclerosis and subsequent vascular complications. In the course of their study, T. S. Slobodchikova et al. [4] revealed that polymorphism of *ADD1, AGT, AGTR, AGTR2, CYP11B2, GNB3* and *NOS3* genes plays a significant role in predisposition to pre-eclampsia. These genes are involved in various physiological processes, including blood pressure regulation, hormone metabolism, and vascular system function. For example, polymorphisms in the *AGT* and *AGTR* genes can affect the activity of the renin-angiotensin-aldosterone system, which can lead to impaired regulation of vascular tone and increased blood pressure, contributing to the development of pre-eclampsia.

In Kazakhstan, where the population is characterised by a unique genetic background and epidemiological features, the study of factors associated with ACVD in pre-eclampsia is of particular interest. Understanding which immune response genes and lipid metabolism genes are associated with an increased risk of developing ACVD in this population may help in the development of more effective prevention and treatment strategies. For example, M. Zh. Espenbetova et al. [5] found that pathological insulin resistance in obesity and physiological insulin resistance lead to inhibition of nitric oxide and prostaglandin synthesis, which causes endothelial dysfunction – a key maternal factor in the development of pre-eclampsia. Endothelial dysfunction can appear long before pregnancy due to chronic diseases such as metabolic syndrome [6,7,8]. Suppression of nitric oxide synthesis, on which platelet aggregation and adhesion, vasodilatory function, vascular wall permeability and vascular cell proliferation depend, plays an important role in the development of pre-eclampsia in pregnant women. Also, A. S. Nurgazina et al. [9] concluded that for labouring women the main risk factors for the development of pre-eclampsia were age, ethnicity, the presence of chronic hypertension or eclampsia in the history, which is also reflected in the study of T. Wainstock et al. [10].

The aim of this article is to analyse genes related to immune response and lipid metabolism and their association with ACVD in pre-eclampsia in the Kazakh population. The objectives of the study include determining the frequency of single nucleotide polymorphisms (SNPs) of genes related to immune response and lipid metabolism in the Kazakh population, identifying the association between genetic markers and the risk of developing ACVD in women with pre-eclampsia, and conducting a comparative analysis of the frequencies of genetic polymorphisms in the Kazakh population with other ethnic groups to assess population-specific risks.

## Materials and Methods

### Study participants

Study participants were provided with information about the purpose of the study to investigate genetic factors associated with pre-eclampsia and ACVD, and procedures for collecting blood samples and medical data were described. They were assured of confidentiality, voluntary participation, the right to withdraw at any time without repercussions, and the potential risks and benefits were explained and then signed a voluntary consent for the use of their blood samples and medical history data. Permission to conduct the study was obtained from the local ethical committee of the Kazakh National Medical University named after S. D. Asfendiyarov (Almaty, Kazakhstan) under application No. 1189 dated 28 September 2021. The study complied with all ethical norms and international standards.

### Control group

The control group included 1,800 conditionally healthy participants of Kazakh ethnicity. The inclusion criteria were belonging to the Kazakh ethnic group (including those over 65 years of age), an age from 18 to 75 years and the ability to make an independent decision to agree to participate in the project. All participants underwent a preliminary questionnaire to confirm that they met these criteria. The authors informed the participants about the anonymous and voluntary participation, and the participants provided their consent. DNA samples were stored in the Miras Biobank of the Scientific Centre of Obstetrics, Gynaecology and Perinatology NAO, established within the framework of the project “Genetic studies of pre-eclampsia in Central Asian and European populations” (InterPregGen) under grant agreement No. 282540 under the 7th Framework Programme of the European Commission.

### DNA isolation

DNA extraction from peripheral blood was performed by magnetic separation of M-PVA particles on a Prepitto automated analyser (PerkinElmer, USA) using the Prepito DNA CytoPure reagent kit (Wallac Finland). This method ensured high purity and concentration of DNA, which was necessary for subsequent genotyping. The isolation process included several stages of purification and concentration, which minimised the risk of contamination and ensured high quality of genetic material.

### Genotyping

Genotyping was performed on 2.5 million SNPs at deCODE Genetics using Illumina Omni Chip 2.5–8 chips as part of the InterPregGen project. This approach provided detailed data on genetic polymorphisms associated with immune response and lipid metabolism. PLINK software was used to control the quality of the Miras Biobank genomic database, and SNPs with minor allele frequency (MAF) less than 1%, call rate below 98%, p<5×10^−7^ and deviation from Hardy-Weinberg equilibrium (*P* < 0.05) were excluded. The application of these criteria ensured high accuracy and reliability of the results obtained.

### Data analysis

Six polymorphisms of genes related to immune response and lipid metabolism were successfully screened for quality standards. The nonparametric χ² test implemented using PLINK software was used for statistical analysis. Genotype frequencies were checked for compliance with the Hardy-Weinberg law (HWE) using the HWE-test function in PLINK software. Statistical significance of the results was determined at the level of *p* < 0.05. Additionally, comparative studies were performed to compare the data of our population with the results of studies of other populations. A variety of databases and results of genome-wide association studies (GWAS) of genetic aspects of pre-eclampsia were used to select significant SNPs associated with acute cerebral circulatory failure. The list of resources used included the National Centre for Biotechnology Information (NCBI), Ensembl, Genotype and Phenotype Database (dbGaP), National Human Genome Research Institute (NHGRI), GWAS catalogue, 1000 Genomes Project and others. These resources have provided extensive information on potential genetic markers and their association with disease.

### Justification for SNP selection

Although initial genotyping encompassed over 2.5 million SNPs using the Illumina Omni Chip 2.5-8 platform, only six SNPs were retained for focused analysis based on stringent filtering and functional relevance. The selection process was guided by both statistical and biological criteria. First, SNPs were filtered for data quality using PLINK software, excluding those with a MAF below 1%, call rate under 98%, and significant deviation from the Hardy-Weinberg equilibrium (*p* < 0.05). Second, among the high-quality variants, candidate SNPs were prioritised based on their known or hypothesised involvement in the pathophysiological pathways of pre-eclampsia and acute cerebral circulatory failure, specifically within the domains of immune response and lipid metabolism. Selection was informed by a comprehensive review of GWAS, meta-analyses and curated databases such as NCBI, Ensembl, NHGRI GWAS Catalog, dbGaP, and the 1000 Genomes Project. The final set included three SNPs related to immune function (*TLR4* rs4986790, *PLEKHA1* rs2281673 and *PLEKHG1* rs9478812) and three related to lipid metabolism (*APOE* rs7412, *FTO* rs1421085, and *LPL* rs285). These SNPs were chosen due to their established or potential mechanistic roles in inflammatory signalling, endothelial dysfunction, lipid dysregulation and metabolic stress – key processes implicated in the aetiology of pre-eclampsia and its vascular complications. Thus, the focused selection enhances biological interpretability, reduces the risk of type I error associated with multiple testing, and aligns with the study’s goal of identifying population-specific genetic risk markers in a Central Asian cohort.

## Results

### Allele and genotype frequencies

A study of SNPs associated with ACVD in pre-eclampsia in the Kazakh population revealed significant differences in the frequencies of minor alleles of genes responsible for immune response and lipid metabolism. The present study has implications for understanding genetic risk factors for the development of pre-eclampsia and its complications in a particular ethnic group.

The study analysed genes associated with immune response and lipid metabolism in the Kazakh population. Immune response genes include *TLR4* (rs4986790), *PLEKHA1* (rs2281673) and *PLEKHG1* (rs9478812). Lipid metabolism genes include *APOE* (rs7412), *FTO* (rs1421085) and *LPL* (rs285). The frequencies of minor alleles in the studied sample of Kazakhs were as follows: for *TLR4* gene (rs4986790) the frequency was 3.3%, for *PLEKHA1* (rs2281673) – 5.9%, for *PLEKHG1* (rs9478812) 27.0%, for *APOE* (rs7412) 7.8%, for *FTO* (rs1421085) 28.3%, and for *LPL* (rs285) the minor allele frequency was at 36.0%.

These data emphasise significant population-specific differences in gene frequencies, which may have important implications for understanding genetic susceptibility to diseases such as pre-eclampsia in the Kazakh population. SNP rs4986790 of *TLR4* gene showed the lowest frequency of minor allele – 3.3%, and rs285 of the *LPL* gene showed the highest – 36.0%. These data indicate the presence of specific genetic characteristics in the Kazakh population that may influence the risk of pre-eclampsia and related complications such as ACVD. It is essential to note that allele frequencies may vary between different ethnic groups, emphasising the need for population-specific genetic studies (Table 1).

**Table 1.** Frequencies of minor alleles of GWAS SNPs associated with acute cerebral circulatory failure in pre-eclampsia in the Kazakh population.

**Gene name**	**r**	**MAF**	**N**	**A1**	**A2**	**GENO**
**Immune response genes**						

*TLR4*	rs4986790	0.033	1801	A	G	1683/117/1
*PLEKHA1*	rs 2281673	0.059	1800	T	A	1589/208/3
*PLEKHG1*	rs 9478812	0.270	1798	G	A	935/727/136

**Lipid metabolism genes**						

*APOE*	rs 7412	0.078	1801	T	C	1531/259/11
*FTO*	rs 1421085	0.280	1798	T	C	932/713/153
*LPL*	rs 285	0.360	1801	T	C	709/867/225

*Note:* rs is the SNP identifier; MAF is the minor allele frequency; N is the number of samples; A1 is the wild-type allele and A2 is the minor allele; GENO is the number of identified genotypes.

*Source:* created by the authors.

The *TLR4* gene encoding Toll-like receptor 4 plays a key role in the activation of the innate immune response. This receptor recognises pathogen-associated molecular patterns (PAMPs) and endogenous damage-associated molecules (DAMPs), initiating an inflammatory response. *TLR4* is expressed on the surface of various cells of the immune system, including macrophages, dendritic cells and neutrophils. Upon activation, *TLR4* triggers a signalling cascade leading to the production of pro-inflammatory cytokines and chemokines.

The low frequency of the minor allele of the *TLR4* gene (3.3%) in the Kazakh population may indicate a reduced probability of excessive inflammatory reactions that may contribute to the development of pre-eclampsia and ACVD. This may be the result of evolutionary adaptation to reduce the risk of autoimmune and inflammatory diseases. However, it may also mean that the body may be less efficient at recognising and responding to certain pathogens or tissue damage, which may have its own negative consequences in the context of other diseases. Low-frequency variants may not have high predictive value for conditions such as pre-eclampsia and SLE due to their rarity. However, they provide valuable information about mechanisms, revealing how genetic variations can influence immune responses and inflammation. In particular, the *TLR4* variant may protect against excessive inflammation, contributing to a better understanding of disease mechanisms, even if it is not a reliable predictor in clinical practice.

In contrast, the high frequency of the minor allele of the *LPL* gene (36.0%) associated with lipid metabolism may indicate a higher predisposition to metabolic disorders in the Kazakh population. Lipoprotein lipase, encoded by the *LPL* gene, plays a pivotal role in lipoprotein and triglyceride metabolism [11,12]. This enzyme hydrolyses triglycerides in chylomicrons and very low-density lipoproteins (VLDL), thereby allowing delivery of fatty acids to tissues for use or storage. Alterations in lipoprotein lipase activity may affect the lipid profile and consequently the risk of cardiovascular disease, including complications of pre-eclampsia [13,14]. The high frequency of the minor allele of the *LPL* gene may be associated with altered enzyme activity, which may lead to abnormalities in lipid metabolism. This, in turn, may contribute to the development of dyslipidaemia, obesity and insulin resistance, factors that play an important role in the pathogenesis of pre-eclampsia and cardiovascular complications.

The *PLEKHG1* gene, which showed a minor allele frequency of 27.0%, is involved in the regulation of cell signalling and cytoskeleton reorganisation. The protein encoded by this gene is a guanine nucleotide exchange factor (GEF) for small GTPases of the Rho family. These GTPases play important roles in a variety of cellular processes including actin cytoskeleton reorganisation, cell adhesion, migration, and polarisation. These processes are important for normal vascular endothelial function, the disruption of which is one of the key mechanisms for the development of pre-eclampsia. The endothelial dysfunction characteristic of pre-eclampsia may be due in part to dysregulation of the cytoskeleton and intercellular contacts, in which *PLEKHG1* is involved [15,16]. The intermediate frequency of the minor allele of this gene (27.0%) may indicate a balance between protective and risk factors in relation to the development of vascular complications in the Kazakh population.

The *PLEKHA1* gene, the minor allele of which has a frequency of 5.9% in the study population, encodes a protein containing a pleckstrin homologous domain. This protein is involved in cell signalling and vesicular transport. Although its exact role in the development of pre-eclampsia and ACVD is not completely clear, alterations in its function may affect intracellular signalling pathways, potentially contributing to pathological processes.

The *APOE* gene encoding apolipoprotein E plays a key role in lipoprotein and cholesterol metabolism. The frequency of minor allele SNP rs7412 of this gene in the Kazakh population was 7.8%. Apolipoprotein E is involved in transport and metabolism of lipids, as well as in the repair and regeneration of nervous tissue. Different *APOE* isoforms (E2, E3, E4) are associated with different risk of cardiovascular and neurodegenerative diseases. In the context of pre-eclampsia and ACVD, variations in the *APOE* gene may influence the lipid profile and susceptibility of the vascular system to damage.

The *FTO* gene, known for its association with obesity and metabolic syndrome, showed a minor allele frequency of SNP rs1421085 28.3% in the Kazakh population. The *FTO* protein functions as a nucleic acid demethylase and is involved in the regulation of metabolism and energy balance. Variations in the *FTO* gene may influence the risk of obesity, which is a known risk factor for pre-eclampsia and cardiovascular complications.

### Compliance with the Hardy-Weinberg law

The distribution of expected and observed genotype frequencies of the genes under study in the Kazakh population was analysed for compliance with the Hardy-Weinberg law. This law describes the stability of allele and genotype frequencies in an ideal population in the absence of evolutionary factors such as mutations, natural selection, gene drift and migration.

The Hardy-Weinberg law is based on several key assumptions. First, the population must be infinitely large, which minimises the effect of random variation in allele frequencies. This condition eliminates the effect of gene drift, which can significantly alter allele frequencies in small populations. Second, mating in the population must occur randomly (panmixia), which means that all individuals have an equal chance to reproduce regardless of their genotype. Third, there should be no natural selection in the population, meaning that all genotypes should have the same fitness and probability of survival and reproduction. In addition, there should be no mutations in the population that could alter allele frequencies. This assumption rules out genetic variability that could arise from new mutations. Finally, for the Hardy-Weinberg law to hold, there must be no migration of genes into or out of the population, which prevents the inflow or outflow of genetic material that could alter allele frequencies. All these assumptions together create ideal conditions under which allele and genotype frequencies remain constant from generation to generation, allowing Hardy-Weinberg’s law to be used to predict population genetic structure in the absence of evolutionary forces.

All the SNPs studied were in Hardy-Weinberg equilibrium (*p* > 0.05), indicating genetic equilibrium in the population. This is an important result, as it confirms that the study sample was representative and was not subjected to significant external influences that could alter allele and genotype frequencies (Table 2).

**Table 2.** Correspondence of genotype distribution to Hardy-Weinberg equilibrium genes associated with the development of acute cerebral circulatory failure in pre-eclampsia in the Kazakh population.

**Gene name**	**rs**	**N**	**GENO**	**O(HET)**	**E(HET)**	**p**
**Immune response genes**						

*TLR4*	rs4986790	1801	1683/117/1	0.06496	0.06389	0.7201
*PLEKHA1*	rs 2281673	1800	1589/208/3	0.1156	0.1118	0.2046
*PLEKHG1*	rs 9478812	1798	935/727/136	0.4043	0.4013	0.7691

**Lipid metabolism genes**						

*APOE*	rs 7412	1801	1531/259/11	0.1438	0.1439	1
*FTO*	rs 1421085	1798	932/713/153	0.3966	0.4061	0.3236
*LPL*	rs 285	1801	709/867/225	0.4814	0.4639	0.1155
*ZNF 831*	rs 6015450	1801	1632/164/5	0.09106	0.09195	0.6064

*Note:* rs is a unique SNP; MAF is the frequency of the minor allele in the study population; N is the total number of individuals studied; GENO indicates the number of reported genotypes; O(HET) is the expected heterozygosity calculated based on the Hardy-Weinberg law; E(HET) is the actual-observed heterozygosity compared with the theoretical Hardy-Weinberg equilibrium; P is the p-value, which shows the statistical significance of the observed data.

*Source:* developed by the authors.

This also indicates the absence of significant evolutionary pressures on the studied genes in the population, which allows for the consideration of obtained frequencies as a basic for the Kazakh population. The data obtained in this study can be used for further genetic studies and comparisons with other populations, which contributes to the expansion of knowledge about the genetic structure and adaptations of different ethnic groups.

### Comparative analysis of allele frequencies

The results of a comparative analysis of minor allele frequencies of genes related to immune response and lipid metabolism in the Kazakh population and in populations of South Asian and European countries revealed significant differences. During the comparison, adjustment for the false discovery rate was used. This method allowed for a more balanced approach, preserving statistical power while minimising the likelihood of false discoveries in large-scale SNP analyses. The detected differences may be due to multiple factors, including geographic isolation, historical migration routes, founder effects, and various evolutionary pressures acting on the populations over time. For example, the frequency of the minor allele SNP rs4986790 of the *TLR4* gene in the Kazakh population was 3.3%. This is significantly lower than in European populations, where this indicator reaches 5.7%, and in South Asian populations, where it is 12.0%. Such differences may indicate possible ethnic variations in genetic susceptibility to inflammatory processes. This, in turn, may influence the risk of developing diseases such as pre-eclampsia and ACVD.

The lower frequency of the *TLR4* minor allele in the Kazakh population may indicate a lower genetic predisposition to an excessive inflammatory response, which may be a protective factor against certain inflammatory diseases. This may be the result of adaptation to certain environmental or pathogenic factors specific to the region. However, it may also mean a reduced ability to recognise certain pathogens, which may have its own negative consequences in the context of infectious diseases.

The frequency of the minor allele SNP rs2281673 of the *PLEKHA1* gene was 5.9%, which is not significantly different from the frequency in South Asian populations (8.2%) and significantly higher compared to European populations (0%). These data suggest a possible role of this gene in the pathogenesis of pre-eclampsia and ACVD in Asian populations, but not in European populations. The *PLEKHA1* gene is involved in the regulation of intracellular signalling and may influence the processes associated with the development of vascular complications. Differences in allele frequencies of this gene between populations may partly explain differences in the prevalence and course of pre-eclampsia in different ethnic groups.

The presence of this minor allele in Asian populations, including the Kazakh population, and its absence in European populations may indicate specific adaptations to environmental or dietary conditions characteristic of the Asian region. It may also indicate different mechanisms of development of pre-eclampsia and its complications in different ethnic groups, highlighting the importance of population-specific studies and personalised approach to diagnosis and treatment.

The minor allele SNP rs9478812 of the *PLEKHG1* gene had a frequency of 27.0% in the Kazakh population, which was significantly lower than in East Asian populations (52.0%) and higher than in European populations (21.0%). These differences may reflect different genetic mechanisms involved in the development of pre-eclampsia and its complications in different ethnic groups. The *PLEKHG1* gene is involved in the regulation of cell signalling and cytoskeleton reorganisation, which is important for normal vascular endothelial function. The intermediate frequency of the minor allele of this gene in the Kazakh population may indicate a unique balance between risk and protective factors with respect to the development of vascular complications. This frequency distribution may be the result of the complex history of the Kazakh population, including the mixing of different ethnic groups. It may also reflect specific adaptations to environmental and social factors characteristic of the region (Table 3).

**Table 3.** Comparative analysis of allele frequencies of immune response and lipid metabolism genes in the Kazakh population of GWAS associated with the development of acute cerebral circulatory failure in pre-eclampsia with populations of the world countries.

**Population**	**N**	**MAF**	***χ* ^2^**	**R**
**Immune response genes**				
***TLR4* (rs4986790)**				

Kazakhstan	1801	0.033		
Europe	503	0.057	4.487	0.035
East Asia	504	0	17.239	<0.001
South Asia	489	0.12	59.549	<0.001

***PLEKHA1* (rs2281673)**				

Kazakhstan	1800	0.059		
Europe	503	0	31.050	<0.001
East Asia	504	0.13	29.601	<0.001
South Asia	489	0.082	3.380	0.066

***PLEKHG1* (rs9478812)**				

Kazakhstan	1798	0.27		
Europe	503	0.21	7.170	0.008
East Asia	504	0.52	112.329	<0.001
South Asia	489	0.28	0.211	0.647

**Lipid metabolism genes**				
***APOE* (rs7412)**				

Kazakhstan	1801	0.078		
Europe	503	0.063	1.134	0.287
East Asia	504	0.10	2.400	0.122
South Asia	489	0.044	6.273	1

***FTO* (rs1421085)**				

Kazakhstan	1798	0.28		
Europe	503	0.43	40.982	<0.001
East Asia	504	0.16	29.463	<0.001
South Asia	489	0.30	0.822	0.365

***LPL* (rs285)**				

Kazakhstan	1801	0.36		
Europe	503	0.48	23.625	<0.001
East Asia	504	0.66	145.862	<0.001
South Asia	489	0.59	85.036	<0.001

*Note:* N – number of DNA samples; MAF – minor allele frequency; χ^2^ – chi-square test; p – statistical significance.

*Source:* compiled by the authors based on 1,000 Genomes Project [17].

Regarding lipid metabolism genes, the frequency of the minor allele SNP rs285 of the *LPL* gene in the Kazakh population (36.0%) was significantly lower compared to the European (48.0%) and East Asian (66.0%) populations. This may indicate a potentially more favourable lipid profile in the Kazakh population, which may serve as a protective factor against the development of ACVD in pre-eclampsia.

Lipoprotein lipase, encoded by the *LPL* gene, plays a key role in lipid metabolism by hydrolysing triglycerides in lipoproteins. Differences in the allele frequency of this gene may influence the efficiency of lipid metabolism and therefore the risk of cardiovascular disease. The lower frequency of the minor allele in the Kazakh population may be associated with adaptation to a traditional diet and lifestyle, potentially reducing the risk of metabolic disorders.

The frequency of the minor allele SNP rs7412 of the *APOE* gene in the Kazakh population was 7.8%, which is not significantly different from the frequencies in European and East Asian populations. The *APOE* gene plays an important role in lipoprotein metabolism and is associated with the risk of cardiovascular disease. The similarity of frequencies of this allele between populations may indicate a universal role of this gene in lipid metabolism and the development of vascular complications.

Apolipoprotein E, encoded by the *APOE* gene, is involved in lipid transport and metabolism and plays a role in neural tissue regeneration. Different *APOE* isoforms (E2, E3, E4) are associated with different risks of cardiovascular and neurodegenerative diseases. The similarity of minor allele frequencies between populations may indicate the conservation of an important functional role of this gene during human evolution.

The frequency of the minor allele SNP rs1421085 of the *FTO* gene in the Kazakh population was 28.3%. The *FTO* gene is known for its association with obesity and metabolic syndrome. Variations in this gene may affect eating behaviour and energy balance of the body. The intermediate frequency of the minor allele in the Kazakh population may reflect a balance between obesity risk factors and adaptation to traditional lifestyle and diet.

### Genetic markers

The results of this study showed that SNPs of immune response genes and lipid metabolism can be used as prognostic genetic markers for the development of pre-eclampsia and its complications in the form of ACVD in the Kazakh population. These markers can be used to develop personalised approaches to the prevention and treatment of pre-eclampsia and its complications.

SNP rs285 of the *LPL* gene, the minor allele of which has a frequency of 36.0% in the Kazakh population, can be used as a protective marker of the risk of ACVD development in pre-eclampsia. Its lower frequency compared with European and East Asian populations may indicate a potentially more favourable lipid profile in the Kazakh population. This may be associated with a lower risk of atherosclerosis and other vascular complications that may aggravate the course of pre-eclampsia and increase the risk of ACVD. The use of SNP rs285 of the *LPL* gene as a genetic marker may help in the risk stratification for the development of ACVD in patients with pre-eclampsia. Patients with a protective allele may have a lower risk of developing severe vascular complications, which may influence the management tactics of pregnancy and labour. The frequencies of other SNPs such as rs7412 of the *APOE* gene and rs1421085 of the *FTO* gene also indicate the possibility of their use as prognostic markers. The *APOE* gene, known for its role in lipid metabolism and cardiovascular disease risk, showed a minor allele frequency of 7.8% in the Kazakh population, which is not significantly different from frequencies in European and East Asian populations. This may indicate a universal role of this gene in the development of vascular complications in pre-eclampsia.

Different *APOE* isoforms (E2, E3, E4) are associated with different risk of cardiovascular disease. Determination of *APOE* genotype may help in assessing individual risk of vascular complications in pre-eclampsia and ACVD. For example, carriers of the E4 allele may have an increased risk of atherosclerosis and other vascular pathologies, which may require more intensive monitoring and preventive measures.

The *FTO* gene, associated with obesity and metabolic syndrome, may also play an important role in the development of pre-eclampsia and its complications. The frequency of minor allele SNP rs1421085 of this gene in the Kazakh population was 28.3%, which may reflect an intermediate risk of metabolic disorders associated with pre-eclampsia. Variations in the *FTO* gene may influence the risk of obesity, which is a known risk factor for pre-eclampsia and cardiovascular complications. Determination of the *FTO* genotype may help to identify patients at increased risk of developing metabolic disorders and hence pre-eclampsia. This may be particularly important for the development of personalised prevention strategies, including dietary recommendations and physical activity programmes.

Pre-eclampsia and acute cerebrovascular disease are complex diseases, indicating that their onset is influenced by both hereditary and several non-genetic factors related to the environment and lifestyle [18,19,20]. These factors include maternal age, comorbidities (including hypertension, diabetes, and obesity), socioeconomic status, and access to healthcare. These factors may interact with genetic predisposition identified in the study, thereby altering the clinical manifestations of pre-eclampsia and ACVD in complex ways. Environmental factors, including dietary habits (e.g., excessive sodium intake or insufficient calcium intake) and lifestyle (e.g., low physical activity, smoking), may exacerbate the influence of genetic risk factors, especially those related to lipid metabolism and immune function. In addition, the presence of comorbidities such as obesity, metabolic syndrome, or chronic hypertension, which are common in populations with an increased genetic predisposition to metabolic disorders, can significantly alter the expression of genetic variants, thereby increasing the risk of developing pre-eclampsia or HELLP syndrome.

The relationship between genes and the environment in relation to pre-eclampsia and HELLP syndrome is important for understanding the etiology of the disease and improving its treatment. Genetic variations in lipid metabolism genes, such as *LPL* or *APOE*, which play a role in lipid homeostasis and vascular function, can be altered by environmental factors such as diet or physical activity, thereby influencing the onset of dyslipidemia and its subsequent vascular consequences. Similarly, immune response genes, such as *TLR4*, may interact with inflammatory environmental stimuli, including infections or stress, thereby exacerbating immune system dysfunction and vascular damage. In addition, access to medical care and prenatal care is crucial in the treatment of these diseases, as timely monitoring and interventions can reduce the risks associated with both genetic predisposition and environmental exposure. Therefore, understanding these relationships is important for developing individualised strategies for the prevention and treatment of pre-eclampsia and HELLP syndrome.

## Discussion

The main findings of the study emphasise the importance of immune response genes and lipid metabolism in the pathogenesis of these conditions. The *TLR4* gene, a minor allele of which occurs with a frequency of 3.3%, attracts the greatest attention. This gene plays a key role in the activation of the innate immune response and inflammatory processes. The low frequency of the *TLR4* minor allele may indicate a reduced likelihood of excessive inflammatory responses, which may be a protective factor against the development of ACVD. However, it may also indicate a reduced ability of the body to recognise certain pathogens, which may lead to other risks. One of the key aspects is to identify the frequencies of minor alleles of *TLR4, PLEKHA1, PLEKHG1, APOE, FTO* and *LPL* genes in the Kazakh population. The data obtained demonstrate significant differences in the frequencies of these alleles compared to other ethnic groups. This indicates unique genetic characteristics of the Kazakh population that may play a role in the development of pre-eclampsia and its complications, such as ACVD.

Recent studies have emphasised the key role of Toll-like receptor 4 (*TLR4*) in the pathogenesis of pre-eclampsia. In a meta-analysis from 2021, M. Sun et al. [21] found that polymorphisms rs4986790 and rs4986791 of the *TLR4* gene are associated with an increased risk of pre-eclampsia. This study also determined the frequency of occurrence of the minor allele of this gene, which was 3.3%. This result is consistent with the data of previous studies, confirming their reliability and indicating the representativeness of the sample. Further, in the study of N. Lind et al. [22] from 2022, the authors showed that *TLR4* activation and cytokine imbalance in monocytes of women with pre-eclampsia contribute to inflammation. These results confirm the important role of the inflammatory process in the development of pre-eclampsia and highlight the importance of *TLR4* in this context. Although the above study focuses on the functional activity of *TLR4* rather than genetic polymorphisms, it confirms the role of *TLR4* in the pathogenesis of pre-eclampsia.

In a review by P. Firmal et al. [23] from 2020, the authors discuss the role of *TLR4* in immunomodulation in normal pregnancy and related disorders such as pre-eclampsia. This study highlights the importance of *TLR4* in the regulation of immune responses in pregnancy and its potential role in the pathogenesis of pre-eclampsia. The study by M. Romão-Veiga et al. [24] from 2020 also confirmed that women with pre-eclampsia have an increased activation of the *TLR4* pathway and an associated cytokine imbalance. These data further emphasise that *TLR4* plays a key role in the inflammatory process associated with pre-eclampsia and may be a target for therapeutic interventions. These studies indicate that regardless of minor allele frequency, functional *TLR4* activity plays an important role in the pathogenesis of the disease. Finally, in a review by A. Katsafanas and C. Bushnell [25] from 2022, the authors indicate that *TLR4* levels in the placenta and blood can serve as promising biomarkers for the diagnosis of pre-eclampsia. These data point out the importance of *TLR4* in the diagnosis of pre-eclampsia and the possibility of its use in clinical practice, which is also supported by the findings from this study, where a high frequency of this gene was also found. Thus, *TLR4* is a critical element in the pathogenesis of pre-eclampsia, and its genetic polymorphisms and expression levels may serve as diagnostic markers and therapeutic targets.

The *LPL* gene responsible for lipid metabolism showed a high frequency of minor allele (36%). This gene encodes a lipoprotein lipase important for lipid metabolism. The high frequency of the minor allele may indicate a predisposition to metabolic disorders, which requires further studies to understand their impact on the risk of ACVD. Studies show that placental *LPL* and CGI-58 levels are significantly reduced in women with pre-eclampsia, which is associated with impaired lipid metabolism and elevated levels of triglycerides, low density lipoprotein (LDL-C) and apolipoprotein B (ApoB), and reduced levels of high-density lipoprotein (HDL-C) and apolipoprotein A (ApoA) [26]. The study included 52 women with pre-eclampsia and 32 women without the condition. However, genetically determined levels of lipoprotein(a) (Lp(a)), despite its role in blood clotting, had no significant effect on the risk of pre-eclampsia. These studies are consistent with the findings, indicating the importance of the *LPL* gene in the pathogenesis of pre-eclampsia and a possible association with disorders of lipid metabolism.

In a study involving 318,922 participants for lipoprotein(a) levels and 5,922 cases of pre-eclampsia, the main analysis showed *p* = 0.562 and secondary analyses also showed no significant results. This precludes the usefulness of using screening for Lp(a) to assess the risk of pre-eclampsia [27]. This result is not consistent with the findings. The inconsistency may be due to the fact that the mentioned study analysed blood levels of lipoprotein(a) [Lp(a)] and their association with the risk of pre-eclampsia, whereas the present study focuses on genetic polymorphisms of the *LPL* gene that affect lipid metabolism. Lipoprotein(a) and lipoprotein lipase (a product of the *LPL* gene) are involved in different aspects of lipid metabolism. The lack of significant results for Lp(a) does not exclude a possible association between *LPL* gene polymorphisms and risk of pre-eclampsia, emphasising the complexity of genetic factors in the disease and the need for further investigation of other lipid metabolism genes. Additional studies have shown that *LPL*, ApoC2 and ApoE concentrations are significantly higher in women with pre-eclampsia. In a study involving 90 women (45 normotensive and 45 with pre-eclampsia), these changes may serve as markers of complications of this condition, leading to worse maternal and foetal outcomes [28]. The findings add to the results of this study, indicating a possible role of elevated blood *LPL* levels in the development of pre-eclampsia, despite its decreased expression in the placenta.

Multiplex biomarker analysis revealed differences between early and late pre-eclampsia. In a study involving 115 women (37 with early pre-eclampsia (EPE), 29 with late pre-eclampsia (LPE) and 49 controls), 47 cardiovascular biomarkers were found to differ between groups. Among them, 42 markers differed between controls and EPE, 28 markers between controls and LPE, and nine markers between EPE and LPE. Biomarkers such as *ST2*, *MMP1*, *MMP3* and *CX3CL1* were uniquely impaired in early pre-eclampsia [29]. Although the *LPL* gene is not directly mentioned in the previously described study, the data highlight the complexity of metabolic alterations in pre-eclampsia and the need to further investigate the various genes and proteins involved in the pathological process. Finally, genetic studies of South African women have shown that carriers of the G1 allele of the *APOL1* gene have an increased risk of developing early pre-eclampsia (OR 2.2, *p* = 0.03). The study included 175 women with pre-eclampsia and 171 controls. Among women with early pre-eclampsia, 49% were carriers of at least one minor allele [30]. Although the *APOL1* gene is different from *LPL*, both genes are associated with lipid metabolism, and the findings among carriers of the G1 allele of *APOL1* indicate the importance of genetic factors in the development of pre-eclampsia in different populations, which was reflected in the results of this study. The frequency of the minor allele of the *PLEKHG1* gene (270%) and its role in the regulation of cell signalling are also noteworthy. This gene is involved in cytoskeleton reorganisation, which is important for the normal functioning of vascular endothelium. Changes in its activity may influence the risk of developing pre-eclampsia.

Also, many other genetic factors play an important role in their development. A study of polymorphisms in the *ATP2B1* gene showed that the G and T alleles rs71454161 and rs73196661, respectively, were significantly more common in patients with eclampsia than in healthy pregnant women. These polymorphisms may serve as potential biomarkers for diagnosis and risk assessment of eclampsia [31]. The finding of the significance of polymorphisms in *ATP2B1*, which is involved in blood pressure regulation through encoding plasma membrane calcium pumps, is consistent with the results of the present study, which emphasise the role of genes affecting vascular tone and pressure, such as *TLR4* and *LPL*, in the development of pre-eclampsia. The association of *AGTR1* gene polymorphisms with eclampsia was also confirmed. In particular, T alleles at rs1799870 and rs52936044 were significantly more prevalent in women with eclampsia, suggesting a possible role of these genetic variations in the pathogenesis of the disease [32]. Although the *AGTR1* gene encoding angiotensin II type 1 receptor was not investigated in this study, the results of H.-L. Xu et al. [33] complement the findings on the importance of genes related to the regulation of blood pressure and vascular tone in the development of pre-eclampsia. Both studies point out the importance of genetic factors in the renin-angiotensin system in the pathogenesis of the disease.

*CYP11B2* gene polymorphisms such as rs4543, rs3802228 and rs104894072 were associated with the development of eclampsia. Differences in the allele and genotype distribution of these polymorphisms between eclampsia patients and controls emphasise their significance in the development and progression of eclampsia. The *CYP11B2* gene encoding aldosteron synthase is involved in mineralocorticoid synthesis and affects water-salt balance and blood pressure. Although this gene was not analysed in this study, its significance in the development of eclampsia is consistent with the results of this study indicating the role of lipid metabolism and vascular regulation genes (*LPL, FTO*) in the pathogenesis of pre-eclampsia. Examination of *ERAP1* and *ERAP2* genes revealed that the C/C variant genotype rs30187 of the *ERAP1* gene is associated with an increased risk of eclampsia, and the *ERAP2* haplotype rs2549796(C)-rs2927609(C)-rs11135484(G) is associated with pre-eclampsia. These findings indicate the importance of aminopeptidases in the pathogenesis of hypertensive disorders of pregnancy [34]. *ERAP1* and *ERAP2* genes are involved in processing peptides for antigen presentation via MHC class I, influencing the immune response. In this study, the *TLR4* gene, also associated with immune response, was investigated. Both studies emphasise the importance of immune mechanisms in the development of pre-eclampsia, confirming the significance of genetic variations in immune system genes.

Finally, mutations in the *PLEKHG5* gene have been associated with various neuromuscular diseases such as Charcot-Marie-Tooth disease and lower motor neuron diseases. These mutations lead to defects in outphagy and signalling pathways, which may contribute to the development of these diseases. Although *PLEKHG5* has not been directly linked to eclampsia, its role in neuromuscular diseases points out the importance of further research in this area [35]. The present study analysed the *PLEKHG1* gene, which is involved in the regulation of cell signalling and cytoskeleton reorganisation, which is important for endothelial function. Although *PLEKHG5* and *PLEKHG1* are different genes, both belong to the pleckstrin-homologous domain family and are involved in similar cellular processes. This may indicate a potential role of similar genes in the pathogenesis of pre-eclampsia and the need for further study. The unique genetic characteristics of the Kazakh population can be used to develop personalised approaches to the prevention and treatment of pre-eclampsia and its complications. Genetic markers, such as SNPs of *TLR4* and *LPL* genes, can help in early detection of women at increased risk of developing these conditions and improve clinical outcomes.

For the clinical validation of genetic markers discovered in this study, a multi-stage approach is needed to ensure the accuracy of genetic associations and their application in clinical practice. The reported SNPs associated with immune response and lipid metabolism (e.g., *TLR4, PLEKHA1, LPL*) should be evaluated in larger independent cohorts to confirm the consistency of the results in other populations. This should include replication studies to confirm that the frequency of minor alleles identified in the Kazakh community is also associated with pre-eclampsia and ACVD in other ethnic groups. In addition, functional studies are needed to elucidate the molecular mechanisms by which these genetic polymorphisms influence disease progression, with a particular focus on their role in immune response and lipid metabolism. Once these genetic markers have been validated in different populations, the next step involves the development of clinical screening tools for early detection and personalised treatment in high-risk groups.

## Conclusions

The study confirms the significant role of genetic factors in the development of pre-eclampsia and related ACVD in the Kazakh population. Genes related to immune response and lipid metabolism were found to play a key role in the pathogenesis of these conditions. The low frequency of the minor allele of the *TLR4* gene (3.3%) may be associated with a reduced likelihood of excessive inflammatory reactions, which is a potential protective factor against ACVD in pre-eclampsia. The high frequency of the minor allele of the *LPL* gene (36.0%) indicates a predisposition to metabolic disorders. Changes in *LPL* gene activity may influence the risk of dyslipidaemia and vascular complications.

Significant differences in the frequencies of minor alleles of the studied genes between the Kazakh population and other ethnic groups were revealed, which emphasises the unique genetic characteristics of the Kazakh population. SNPs of immune response and lipid metabolism genes can be used as prognostic markers for early identification of women at increased risk of developing pre-eclampsia and its complications, which contributes to the development of personalised approaches to prevention and treatment. This study showed that genes related to lipid metabolism, such as *LPL*, play a significant role in the development of pre-eclampsia. A high frequency of the minor allele of the *LPL* gene indicates a predisposition to metabolic disorders, which may influence the risk of vascular complications.

The results of this study may improve clinical outcomes in patients with pre-eclampsia in the Kazakh population. Genetic markers, such as SNPs of *TLR4* and *LPL* genes, may help in developing individualised strategies for pregnancy management and prevention of ACVD, which will ultimately reduce maternal and perinatal morbidity and mortality. Thus, the study highlights the importance of genetic analysis and personalised approaches in the management of patients with pre-eclampsia, given the specific genetic features of the Kazakh population. This opens new possibilities for the diagnosis and treatment of pre-eclampsia, as well as for the development of preventive measures, which is an important step in improving maternal and child health.

Despite the findings, this study has a number of limitations that need to be considered when interpreting the data and planning further research. The analysis focused on a limited number of genes and polymorphisms related to immune response and lipid metabolism, which may not cover all genetic factors that influence the development of pre-eclampsia and its complications. The influence of external factors such as lifestyle, nutrition, environmental conditions and socioeconomic status, which may interact with genetic factors, were not considered. Limited sample size, especially when analysing rare alleles, may reduce statistical power and increase the likelihood of random errors, distorting true associations.

This study may help to develop methods for early identification of women at risk of pre-eclampsia and its complications based on genetic analyses. This will personalise prevention and treatment approaches, reduce mortality and improve maternal and child health. The findings can also be used for further studies in other populations to better understand how genetics influences the development of these conditions.
